# A Spacetime Odyssey of Neural Progenitors to Generate Neuronal Diversity

**DOI:** 10.1007/s12264-022-00956-0

**Published:** 2022-10-10

**Authors:** Mengmeng Ge, Amirhossein Sheikhshahrokh, Xiang Shi, Yu-Hong Zhang, Zhiheng Xu, Qing-Feng Wu

**Affiliations:** 1 State Key Laboratory of Molecular Development Biology, Institute of Genetics and Developmental Biology, Chinese Academy of Sciences, Beijing, 100101 China; 2 University of Chinese Academy of Sciences, Beijing, 100101 China; 3 Center for Excellence in Brain Science and Intelligence Technology, Chinese Academy of Sciences, Beijing, 100101 China; 4 Beijing Children’s Hospital, Capital Medical University, Beijing, 100045 China

**Keywords:** Neuronal diversity, Neural progenitors, Spatiotemporal patterning, Cascade diversifying model

## Abstract

To understand how the nervous system develops from a small pool of progenitors during early embryonic development, it is fundamentally important to identify the diversity of neuronal subtypes, decode the origin of neuronal diversity, and uncover the principles governing neuronal specification across different regions. Recent single-cell analyses have systematically identified neuronal diversity at unprecedented scale and speed, leaving the deconstruction of spatiotemporal mechanisms for generating neuronal diversity an imperative and paramount challenge. In this review, we highlight three distinct strategies deployed by neural progenitors to produce diverse neuronal subtypes, including predetermined, stochastic, and cascade diversifying models, and elaborate how these strategies are implemented in distinct regions such as the neocortex, spinal cord, retina, and hypothalamus. Importantly, the identity of neural progenitors is defined by their spatial position and temporal patterning factors, and each type of progenitor cell gives rise to distinguishable cohorts of neuronal subtypes. Microenvironmental cues, spontaneous activity, and connectional pattern further reshape and diversify the fate of unspecialized neurons in particular regions. The illumination of how neuronal diversity is generated will pave the way for producing specific brain organoids to model human disease and desired neuronal subtypes for cell therapy, as well as understanding the organization of functional neural circuits and the evolution of the nervous system.

## Introduction

The complexity and function of the nervous system relies on the generation of unparalleled neuronal diversity across molecular, morphological, functional, and connectional features throughout the developmental continuum. Although the number of neuronal subtypes in the nervous system is still unclear, the emergence of single-cell technology has led to an explosive expansion in our understanding of neuronal diversity [1, 2]. Nevertheless, the origin of neuronal diversity remains a fascinating and fundamentally important question for neuroscientists.

In the developing nervous system, neural progenitor cells (NPCs) first divide symmetrically to expand the neural progenitor pool and then frequently undergo asymmetric division to produce intermediate progenitor cells (IPCs), which further generate postmitotic neurons with potential cell-fate plasticity [3, 4]. After terminal differentiation of neuronal precursors, the neurons ultimately establish their identity and have a stable structure, connections, and functions. It is widely accepted that critical fate decisions that establish the identity and diversity of neurons occur at the progenitor level [5], while emerging evidence demonstrates that environmental and wiring factors coordinate to reshape neuronal identity and contribute to further diversification at the postmitotic level [6–9].

The vertebrate nervous system is primarily subdivided into five brain vesicles during early development: telencephalon, diencephalon, mesencephalon, metencephalon, and myelencephalon. Although previous studies on the origin of neuronal subtypes have revealed the neurogenic development of the cerebral cortex from the telencephalon, of the retina from the optic cup in the diencephalon, and of the spinal cord from the caudal myelencephalon, there is no unified principle governing the generation of neuronal diversity across brain regions. Neurogenesis in the cerebral cortex is mainly driven by a temporal patterning program, whereas the cardinal feature of neurogenesis in the spinal cord is the spatial patterning of neural progenitors along the dorsoventral (D-V) axis [10–12]. It is worth noting that the spatial coding of progenitors may be coordinated with the temporal dynamics of competence to precisely control neuronal production in most scenarios. Here, we first introduce three general strategies employed by NPCs to generate diverse neuronal subtypes, and further review the neurogenic processes instructed by spatiotemporal codes of neural progenitors and multiple mechanisms for neuronal precursors.

## Distinct Strategies for Generating Neuronal Diversity

Neural progenitors appear to adopt multiple strategies to produce distinct neuronal subtypes during lineage specification, rather than employing a single, uniform strategy for generating neuronal diversity. Regardless of the molecular programs specifying neuronal fate, here we focus on three general strategies found within the vertebrate nervous system: the predetermined model, the stochastic model, and the cascade-diversifying model. These models may display region specificity and context-dependent variation, but cooperate with each other to drive the evolution of neural structure and function.

## Predetermined Strategy

**Fig. 1:**
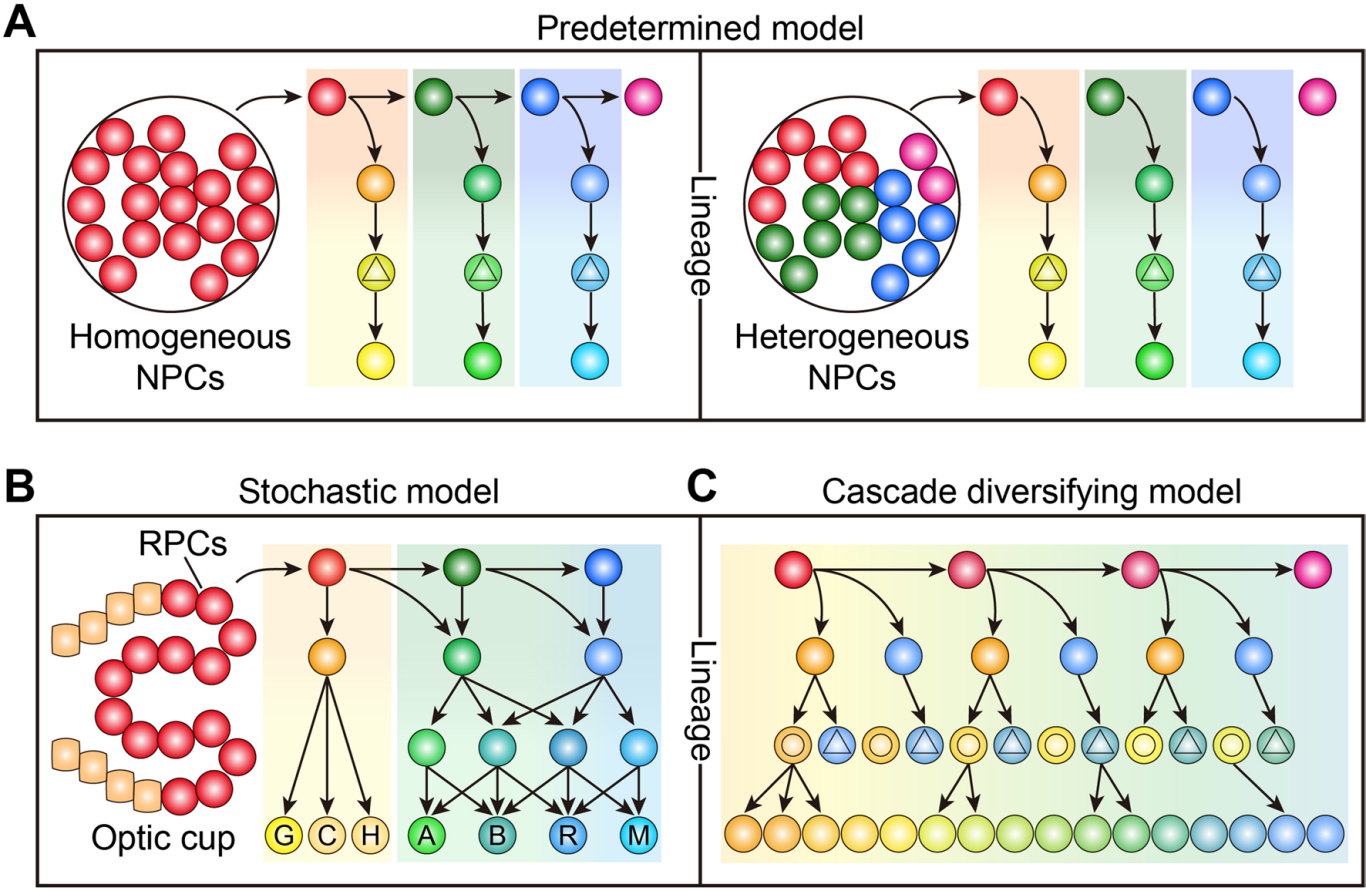
Distinct strategies to produce neuronal diversity. **A** In the predetermined model, homogeneous multipotent (left panel) or heterogeneous fate-restricted NPCs (right panel) in the cerebral cortex sequentially produce subcortical and callosal projection neurons, which divide symmetrically to give rise to postmitotic neurons. **B** In the stochastic model, homogeneous RPCs stochastically produce different types and numbers of cells for the retina throughout their life at the single-cell level. G, retinal ganglion neurons; C, cone cells; H, horizontal cells; A, amacrine cells; B, bipolar cells; R, rod cells; M, Müller cells. **C** The cascade diversifying model suggests that multiple cell types along the lineage hierarchy, including RGCs, IPCs, and nascent neurons, contribute to the fate diversification of neurons in a step-by-step amplifying fashion.

The predetermined model is characterized by the fate specification of neural progenitors into an invariant neuronal subtype within a specified time window. Most of our understanding of this model comes from studies of the neurogenic process in mammalian cerebral cortex, comprised of up to ~80% of excitatory projection neurons and ~20% of GABAergic inhibitory neurons [5, 13, 14]. It is well-established that cortical excitatory neurons are generated in an inside-out manner, organized into six layers and simply classified into two major types: early-born subcortical projection neurons residing in deep layers and late-born callosal projection neurons occupying upper layers [15]. Currently, there are two prevailing hypotheses explaining the specification and diversification of neuronal fates in cerebral cortex [5, 16]. The first hypothesis suggests that homogeneous multipotent NPCs sequentially produce subcortical and callosal projection neurons in a temporal order that occurs *via* fate-predetermined IPCs to a large extent. At a specific time, either the early or late embryonic stage, the fate of endogenous NPCs is programmed and predetermined, but they progressively become fate-restricted (Fig. 1A). The second hypothesis posits the existence of heterogeneous NPCs that are initially fate-predetermined without temporal dynamics. In this case, neural progenitors specifying either subcortical or callosal projection neurons mix with each other in the cortical primordium and are sequentially activated for neuronal production during brain development (Fig. 1A). Albeit many results from transplantation experiments, retroviral lineage tracing, genetic fate mapping, and *in vivo* clonal analysis point toward the hypothesis of multipotent progenitors with strict temporal coding [17–21], it is still possible that a majority of multipotent NPCs coexist with a minority of fate-specified progenitors that remain to be identified. More importantly, both hypotheses support the predetermined model in which the fate of cortical progenitors is pre-specified to produce projection neurons by either a temporal code or an inherent genetic program.

The cortical interneurons are generally classified into three major types based on the expression of parvalbumin (PV), somatostatin (SST), and serotonin receptor 3a (Htr3a), and can be further subdivided into over 20 subtypes [22]. Given the enormous diversity of cortical GABAergic interneurons, whether their fates are also predetermined at the progenitor stage remains controversial. Neural progenitors specifying cortical interneurons predominantly reside in the medial and caudal ganglionic eminences (MGE and CGE) in the ventral telencephalon, which is spatially segregated from dorsal part giving rise to projection neurons. Similar to the origins of projection neuronal subtypes, the emergence of cortical interneuron diversity relies on the coexistence of both multipotent and fate-predetermined NPCs in ganglionic eminences. Compelling evidence has demonstrated that the MGE and adjacent preoptic region produce virtually all PV and SST neurons, while the CGE generates neuronal subtypes that belong among the Htr3a interneurons [23–25]. These reports suggest that neural progenitors with distinct spatial codes are fate-predetermined and have different lineage potentials. In contrast, clonal analysis has revealed the production of both PV and SST interneurons by individual NPCs in the MGE, supporting the multipotent progenitor hypothesis [26, 27]. Further studies have shown that these multipotent progenitor cells give rise to a cohort of globus pallidus neurons, followed by SST and PV interneurons in a temporal sequence [24, 28]. Interestingly, recent single-cell transcriptomic analyses of mouse ganglionic eminences at early embryonic stages indicate that the interneuron identity is already predetermined at the progenitor level or shortly after becoming postmitotic [29, 30]. These results again implicate the fate pre-specification of multipotent progenitors by a temporal code that is very likely to occur at the IPC level [13].

Collectively, we speculate that neural progenitors located in both the dorsal and ventral telencephalon employ the predetermined strategy to generate neuronal diversity, acting through temporal, spatial, or other intrinsic programs. In other words, multipotent progenitors coded by temporal factors produce one neuronal subtype in a specific period, whereas the cellular output of fate-restricted progenitors coded by spatial or intrinsic factors is limited to a single neuronal subtype.

It is notable that the proposition of the classical predetermined model, regardless of progressive competence-restriction or early fate-restriction mechanisms, relies on the simple classification of cortical projection neurons into upper- and deeper-layer neurons. However, the emergence of single-cell RNA sequencing technology provides unprecedented resolution to identify different neuronal subtypes in the cerebral cortex. With the scaled-up resolution of cell taxonomy, the predetermined model only provides a framework for generating neuronal diversity but cannot fully interpret the production of diverse cortical projection neurons at the progenitor level. For example, deep-layer neurons have recently been partitioned into two subtypes that diverge at the postnatal stage [31], suggesting the identity diversification of neurons at the postmitotic level. Moreover, the subdivision of PV interneurons into basket, chandelier, and translaminar cells and the partition of SST interneurons into Martinotti, non-Martinotti, and long-range cells prompted us to propose a more precise model [13, 30]. Given that neuronal diversification occurs at both the progenitor and postmitotic levels, revisiting and refining the predetermined model from the new perspective of cell taxonomy is required in the near future.

## Stochastic Strategy

Although cell fate specification is strictly programmed to generate a highly reproducible organ size and shape, stochastic fate choices are frequently integrated into developmental programs to generate cell fate diversity [32]. In stochastic models, cell fate resembles quantum particles existing in a superposition of states, each occurring with different probabilities until direct observation restricts the probable outcomes to one state (Fig. 1B). The vertebrate retina is a well-characterized model in which to study deterministic or stochastic choices in cell fate specification, due to the limited number of cell types and amenability to observation. There are seven major cell types in the retina: ganglion neurons, horizontal cells, bipolar cells, amacrine cells, Müller cells, cone cells, and rod cells, which are generated by multipotent retinal progenitor cells (RPCs) [33, 34]. Pioneering retroviral lineage tracing in mouse retina revealed that individual RPCs produce multiple cell types with great variability in clonal size (i.e., cell number in clones) and composition (i.e., cell type proportion in clones) [35]. More recent studies using *in vivo* live imaging of mosaically-labeled progenitor cells have shown the stochasticity of zebrafish RPCs in cell division mode and cell production capacity during lineage specification [36, 37]. These multipotent RPCs are seemingly homogenous but produce different numbers and types of cells throughout their life at the single-cell level. Mechanistically, they are subjected to stochastic expression of the key transcription factors required for making a cell fate decision: (1) undergoing symmetric or asymmetric division, (2) producing one of the retinal cell types, and (3) exiting from stemness for terminal differentiation [36, 37]. Although a simple mathematical model has been built to predict the distribution of division mode and clonal size at different developmental time points [36], it is still difficult to understand how the stochastic fate choices of RPCs ultimately give rise to a retina of invariant size and with consistent proportions of cell types.

It is a long-lasting debate whether a stochastic model is compatible with the precise programming of brain development. Interestingly, recent single-cell analyses have identified two types of lineage-specific RPC, each defined by specific transcription factors and producing different sets of retinal neuronal subtypes [38]. In more detail, onecut^+^ RPCs stereotypically produce retinal ganglion neurons, GABAergic amacrine cells, and photoreceptors, whereas vsx1^+^ RPCs specifically differentiate into glycinergic amacrine cells and bipolar cells. This provides a predetermined component for stochastic models. More deterministic factors will be progressively uncovered for the stochastic framework of neurogenesis by multi-omics analysis, lineage tracing, and genetic manipulation.

Coincidentally, stochastic fate decisions also occur in adult stem cell niches to maintain the homeostasis of tissues without expansion or shrinkage. For example, intestinal stem cells make their own stochastic choices to terminally differentiate or expand in adult intestinal crypts, but achieve a delicate balance *via* neutral competition [39, 40]. However, such competition between stem/progenitor cells for limited space and signals may not enable a growing primordium to develop into a tissue or organ with invariant size and shape, especially when the fate choices of progenitor cells are much more complicated than a simple combination of symmetrical differentiation and symmetrical renewal. Thus, future studies will have to determine the mechanism underlying how the stochastic fate specification of neural progenitors builds an invariant tissue. A sophisticated mathematical model is required to connect stochastic factors, deterministic factors, and feedback mechanisms for developing the well-organized cytoarchitecture of neural tissue *in silico*.

## Cascade Diversifying Strategy

In the cascade diversifying model, multiple cell types including neural progenitors and neuronal precursors along the lineage hierarchy contribute to neuronal fate diversification in a stepwise manner (Fig. 1C). This model has recently been proposed to interpret the developmental origin of neuronal diversity in the hypothalamus [41], which arises from the ventral diencephalon and contains an extreme heterogeneity of neurons that control endocrine, autonomic, and behavioral functions. Although we still do not have a satisfactory answer to how many neuronal subtypes there are in the vertebrate hypothalamus, recent unbiased mapping of neuronal identity by single-cell transcriptomic analysis has revealed at least 62 neuronal subtypes with different neurotransmitters and neuropeptides [42, 43]. Given the astounding diversity of neurons in the hypothalamus, it is an ideal model in which to interrogate the developmental mechanism of neuronal subtype generation.

At the progenitor stage, hypothalamic NPCs lining the third ventricle are also supposed to be multipotent and homogeneous but can be subdivided into multiple cell states distinguished by either cell cycle gene sets or distinct transcriptional priming programs [41,44]. Importantly, these neural progenitors give rise to two major groups of IPCs with different transcriptional profiles, lineage specification, and spatial distribution, which are each marked by the transcription factors Ascl1 and Neurog2 [41]. It is notable that Neurog2 expression is restricted to the dorsal telencephalon, whereas Ascl1 is predominantly expressed in the ventral telencephalon, implicating the fate restriction of NPCs in either the dorsal or ventral telencephalon to produce a single group of IPCs [45]. In striking contrast, Ascl1^+^ and Neurog2^+^ IPC domains in the hypothalamus coexist in a mosaic but mutually exclusive pattern. Moreover, it has previously been shown that the proneural factors Ascl1 and Neurog2 direct the differentiation of progenitor cells into neuronal subtypes with different neurotransmitter identities, specifying GABAergic and glutamatergic neurons, respectively, in the telencephalon [46–48]. Nevertheless, a combination of single-cell analysis and lineage tracing has surprisingly revealed that hypothalamic Ascl1^+^ IPCs can differentiate into both GABAergic and glutamatergic neurons, while Neurog2^+^ IPCs exclusively generate glutamatergic neurons [41]. These results support the conclusion that both NPCs and Ascl1^+^ IPCs, residing in the top two tiers of neural lineage hierarchy, are bipotent in neuronal specification.

At the postmitotic stage, the fate of hypothalamic nascent neurons is potentially flexible to be restricted, bifurcated, or multifurcated during their identity establishment. Indeed, genetic fate mapping has indicated that neuronal precursors at a nascent state can resolve into multiple subtypes to amplify their fate diversity [41]. Whether the diversification of neuronal precursors is deterministic or stochastic remains unknown, but it is likely that different groups of neuronal subtypes are genetically segregated and each group shares common neuronal precursors. For example, neuronal subtypes expressing oxytocin, vasopressin, and corticotropin-releasing hormone are organized into a group and may collectively descend from Sim1^+^ nascent neurons [43]. Albeit the neuronal precursors in the developing hypothalamus have been putatively segregated into 29 sublineages for further diversification [41], how neuronal sublineage segregation is organized and the mechanism underlying further neuronal fate choices remain unclear. Therefore, our understanding of the developmental process of hypothalamic neuron diversification is still at an early stage.

The cascade diversifying model is largely built upon the cellular lineage hierarchy at the population level [41]. Despite the fact that clonal analysis has provided proof-of-concept evidence for demonstrating fate multifurcation of individual NPCs, systematic analysis of progenitor cell fate at single-cell resolution is still lacking. It is also unclear how deterministic and stochastic factors shape the cascade diversifying trajectory during lineage progression. Single-cell lineage tracing by integrating an inducible barcoding approach with transcriptomic data may be able to systematically record the developmental trajectory of each individual NPC [21].

Overall, predetermined, stochastic, and cascade diversifying strategies differ from one another in the cellular mechanisms by which they generate neuronal diversity, and in the degree to which cell fates are specified by spatial, temporal, intrinsic, or stochastic factors. Although each of these strategies may dominate in different brain regions to direct neuronal lineage progression, they could orchestrate with each other to generate cellular diversity and build an invariant nervous system in a loosely programmed way with delicate positive or negative feedback mechanisms.

## Spatial Origin of Neuronal Diversity

Spatial partitioning of neural progenitors throughout the five brain vesicles leads to an extensive diversification of progenitors and progeny cells. During early neurodevelopment, the neural tube is patterned along the anteroposterior (A–P) axis by morphogenetic gradients including fibroblast growth factors (FGFs) and retinoic acid and segmented by the Hox gene family [12, 49–51]. Within each segment, neural progenitors are further partitioned along the D–V axis into different progenitor domains, which are shaped by opposing signaling gradients of Wnt/BMP from the roof plate and Shh from the floor plate [52–54]. These signaling gradients counteract each other and confer a spatial code on neural progenitors by inducing the expression of distinct spatial transcription factors (sTFs) [55]. In specific scenarios, cross-repressive interactions between sTFs expressed in neighboring neural progenitor subpopulations establish precise boundaries between different progenitor domains, each of which subsequently produces different neuronal subtypes [56–58]. Thus, the spatial organization of neural progenitors along the A–P and D–V axes allows for their exposure to different morphogen gradients that trigger the establishment of positional identity. Two cellular mechanisms, self-enforcement and cross-repression, may further sculpt the identity of neural progenitors for precise neuronal production.

**Fig. 2:**
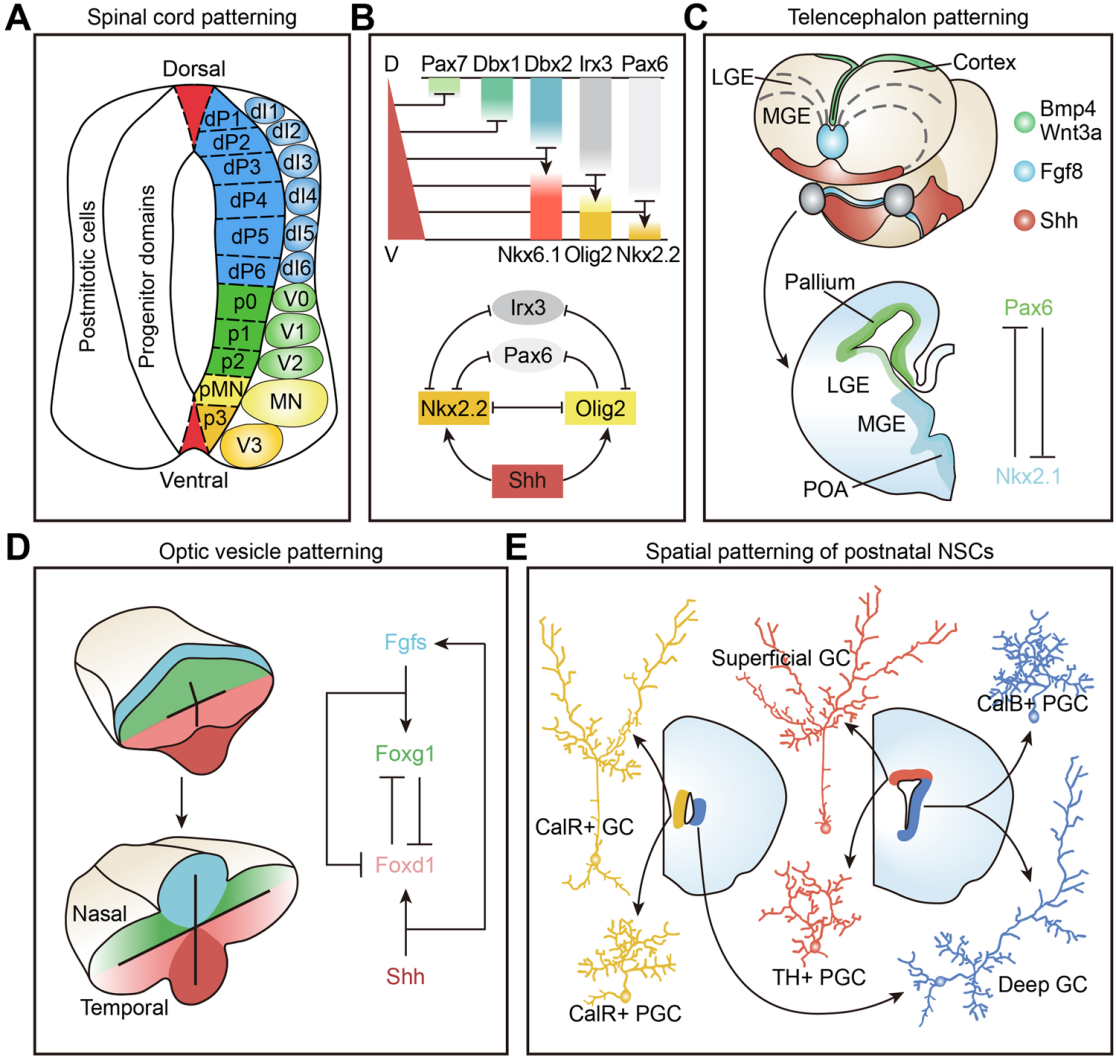
Spatial patterning of neural progenitors for generating neuronal diversity. **A** Spatial compartmentalization of neural progenitors and their descendant neurons in the developing spinal cord along the D–V axis. **B** Graded Shh signaling represses the expression of class I factors (Pax7, Dbx1, Dbx2, Irx3, and Pax6) but induces the activation of class II factors (Nkx6.1, Olig2, and Nkx2.2) at different threshold concentrations. In the ventral spinal cord, increasing levels of Shh signaling lead to the sequential induction of Olig2 and Nkx2.2. **C** Spatial patterning of the telencephalon by Bmp4, Wnt3a, Fgf8, and Shh induces the expression of Pax6 in the dorsal and Nkx2.1 in the ventral forebrain. **D** Morphogen signaling through Fgfs and Shh causes the mutually exclusive expression of Foxg1 and Foxd1 in the nasal and temporal optic vesicle that develops into the retina. **E** In the postnatal brain, dorsal NSCs lining the lateral ventricles generate superficial GCs and dopaminergic PGCs, ventral NSCs produce deep GCs and calbindin^+^ (CalB^+^) PGCs, and medial NSCs give rise to calretinin (CalR)-expressing GCs and PGCs.

In the developing nervous system, the embryonic spinal cord represents one of the best-characterized regions with distinct progenitor domains arrayed along the D–V axis. To be more specific, neural progenitors in the developing spinal cord acquire distinct positional identities in response to opposing morphogen gradients and are organized into six dorsal (dp1–dp6) and five ventral (p0–p2, pMN, and p3) progenitor domains [12, 55]. Each of these domains gives rise to distinct neuronal subtypes, which are classified into 12 groups (dI1–dI6, V0, V1, V2a, V2b, MN, and V3) and distinguished by specific transcription factors (Fig. 2A) [55]. Such spatial patterning of the spinal cord primordium raises the question of how a morphogen gradient is converted into distinct positional identities of neural progenitors. Interestingly, Shh signaling has been reported to specify different progenitor domains by repressing or activating a set of homeodomain transcription factors in a concentration-dependent manner [54, 57]. These homeodomain factors can be subdivided into two classes based on their mode of regulation by Shh: the expression of class I factors (Pax7, Dbx1, Dbx2, Irx3, and Pax6) and class II genes (Nkx6.1, Olig2, and Nkx2.2) is repressed or activated by different Shh threshold concentrations, respectively [58, 59]. Furthermore, four transcription factors (Irx3, Pax6, Nkx2.2, and Olig2) differentially expressed in the p2, pMN, and p3 progenitor domains display cross-repressive interactions to sharpen the gene expression boundaries, with Nkx2.2 and Olig2 repressing the other two factors, Irx3 repressing Nkx2.2 and Olig2, and Pax6 repressing Nkx2.2 (Fig. 2B) [12, 56, 57]. Even though a complete understanding of the gene regulatory network among all these patterning factors has not been reached, the morphogen gradient-mediated transcriptional circuit is supposed to underpin the spatial patterning of the neural tube.

In the mammalian telencephalon, morphogen gradients such as Shh and FGF8 also control the spatial patterning of neural progenitors and induce the expression of sTFs (e.g., Pax6, Emx1, Gsh1/2, and Nkx2.1) that define the positional identity of progenitor cells [60–62]. The combinatorial expression of these sTFs subdivides the telencephalon into four dorsal (medial, dorsal, lateral, and ventral pallium) and three ventral [lateral ganglionic eminence (LGE), MGE, and CGE] progenitor domains along the D–V axis (Fig. 2C). In contrast to the spinal cord, these spatial factors seem not to cross-repress one another to establish distinct progenitor domains in the telencephalon. Similarly, the morphogen signal FGF8 has been found to determine the spatial patterning of zebrafish retina along the nasotemporal (N–T) axis, and prompts the RPCs to adopt a nasal identity by inducing Foxg1 expression (Fig. 2D) [63]. The forkhead transcription factor Foxg1 displays a complementary expression with Foxd1, neither of which establish the molecular identity of progeny cells during RPC differentiation, but coordinate to shape the connectional identity of retinal ganglion neurons [64, 65].

Moreover, the spatial encoding of neural stem/progenitor cells for generating neuronal diversity persists into adulthood. In the postnatal and adult rodent brain, neural stem cells (NSCs) positioned along the lining of the lateral ventricles give rise to neuroblasts that travel *via* the rostral migratory stream into the olfactory bulb and differentiate into different subtypes of interneuron. These adult-born interneurons are divided into two principal types, granule cells (GCs) and periglomerular cells (PGCs), but they exhibit considerable phenotypic diversity with different neurotransmitters, positions, morphology, and connections. Importantly, the origin of different subtypes of interneuron in the olfactory bulb are spatially encoded: dorsal NSCs produce GCs for the superficial layer and dopaminergic PGCs, ventral NSCs yield GCs for the deep layer and calbindin^+^ PGCs, and medial NSCs generate calretinin-expressing GCs and PGCs (Fig. 2E) [66–68]. These studies suggest that the diversity of neurons, regardless of their birth date, is often encoded by the spatial position of neural stem/progenitor cells, and adult NSCs can inherit the spatial ‘heritage’ from residual embryonic NPCs skipping from terminal differentiation.

## Temporal Origin of Neuronal Diversity

It has long been known that the temporal patterning of neural progenitors drives the diversification of neuronal subtypes during the development of the nervous system, which occurs at least in the cerebral cortex and retina [69, 70]. At the onset of neurogenesis in the cerebral cortex, NPCs in the dorsal telencephalon have the competence to generate neurons for all cortical layers and undergo asymmetric division to first produce subcortical projection neurons for the deep layers, but change their competence as development progresses and then produce callosal projection neurons for the upper layers [3, 5, 10, 20]. Similarly, neural progenitors in the ventral telencephalon give rise to SST interneurons at an early stage, whereas PV neurons are generated at a relatively constant rate throughout neurogenesis, corroborating the progressively restricted competence of neural progenitors to generate different subtypes of neuron [13, 22, 24]. In the retina, multipotent RPCs sequentially generate all seven cell types in an overlapping chronological order: they produce early-born cell types including retinal ganglion neurons, horizontal cells, cone cells, and amacrine cells, and then switch to generate late-born cell types like rod cells, bipolar cells, and Müller cells [35, 71, 72]. These reports consistently support the hypothesis that early neural progenitors are multipotent but become progressively restricted in their competence during development. However, whether the temporal generation of different neuronal subtypes is driven by intrinsic mechanisms or extrinsic cues remains to be clarified.

**Fig. 3:**
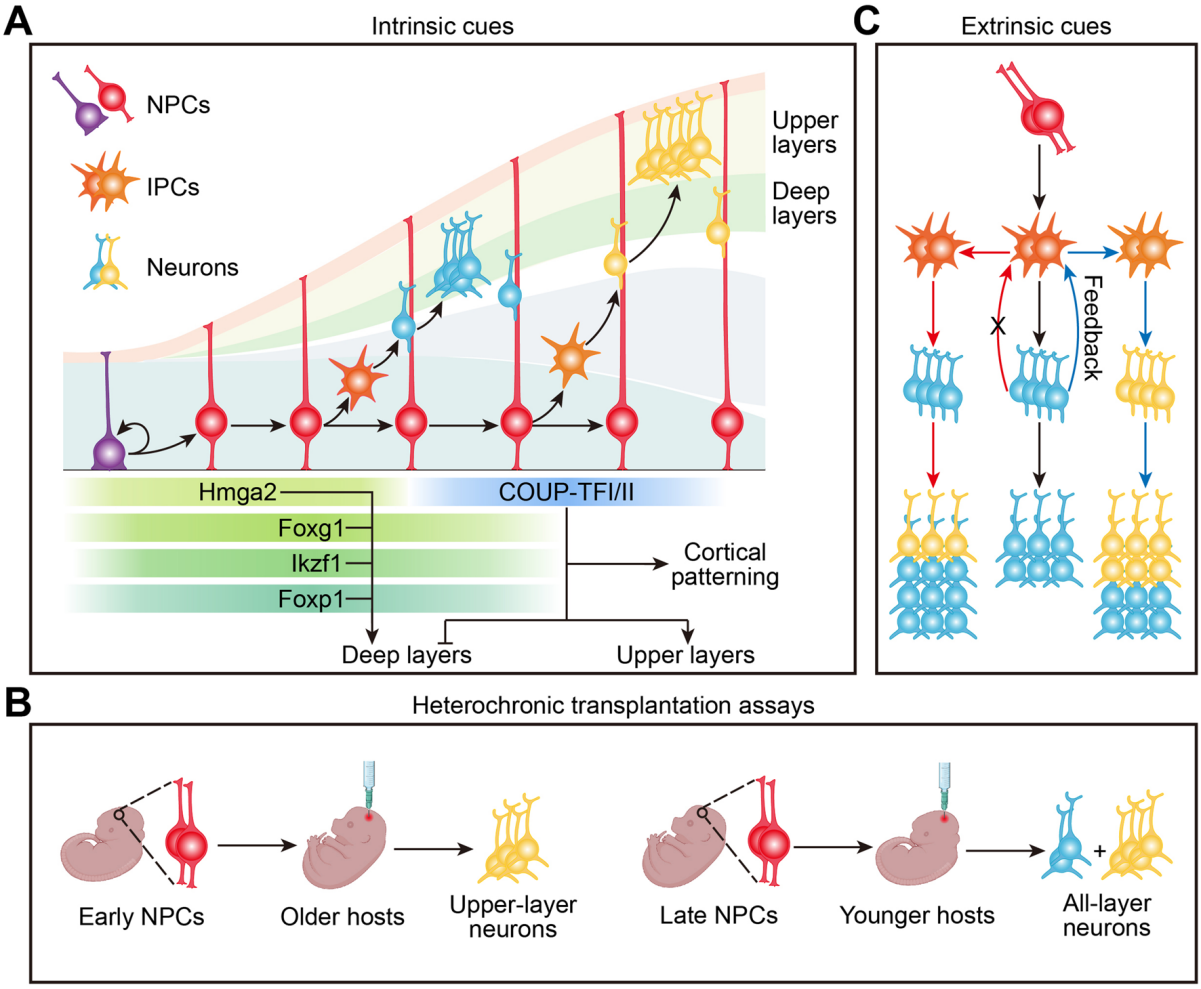
Temporal patterning of neural progenitors to generate neuronal diversity. **A** Temporal coding of neural progenitors in cerebral cortex by a series of tTFs. The TFs Hmga2, Foxg1, Ikzf1, and Foxp1 define the early NPCs and promote the generation of early-born neurons, whereas COUP-TFI/II expressed in late NPCs drive the production of late-born neurons. **B** Schematics showing the fate-restriction of early NPCs to generate upper-layer neurons when transplanted into older hosts, and the fate-reversion of late NPCs to produce neurons for all layers when transplanted into younger hosts. **C** A feedback mechanism that regulates the fate of neural progenitors and the timing transition of neuronal production.

An intrinsic temporal code, defined by temporal cascades of transcription factors or epigenetic factors, has been suggested to choreograph the fate specification of neural progenitors along the developmental timeline. While a recent study has systematically identified a complete series of temporal transcription factors (tTFs) that sequentially specify neuronal fate in the *Drosophila* nervous system and reciprocally regulate each other [73], the tTFs code in the vertebrate nervous system remains unclear. In the developing cerebral cortex, a set of transcription factors such as Foxg1, Ikzf1, Foxp1, and Hmga2 is expressed in early NPCs and promotes deep-layer neuron production and suppresses the generation of late-born upper-layer neurons [74–77]. In contrast, cortical patterning factors COUP-TFI/II are required for the transition from generating deep-layer neurons to upper-layer neurons [78, 79], suggesting the involvement of these factors with temporal dynamics in predetermining neuronal fate (Fig. 3A). In the vertebrate retina, a series of temporal factors including Ikzf1, Pou2f1/2, Foxn4, Casz1, and Nfia/b/x are sequentially expressed in early-, mid-, and late-stage RPCs, and their transcriptional change over the developmental timeline acts as a critical temporal modulator to increase the probability of RPCs to adopt certain cell fates [80–83]. Recently, the application of single-cell technology has greatly facilitated the identification of temporally shifted patterning genes in neural progenitors from the cerebral cortex, retina, and spinal cord [10, 83, 84]. At the same time, it is notable that the tTFs identified in *Drosophila* neuroblasts play much more important roles in specifying neuronal fate than the temporal patterning genes expressed in mammalian neural progenitors. The absence of a given tTF in the fly frequently results in a virtually complete loss of the neuronal fates it controls, whereas genetic ablation of a temporal regulator in the mammalian nervous system only modestly changes the fate specification of early-born or late-born cells, implicating the emergence of redundant regulatory systems that control temporal patterning during evolution.

Progressive changes of extrinsic cues in the neurogenic niches have also been proposed to drive the temporal progression of neural progenitors in cerebral cortex. Heterochronic transplantation assays in ferrets and mice have shown that early NPCs transplanted into older hosts become fate-restricted to produce late-born upper-layer neurons, while late NPCs transplanted into younger hosts reset their temporal state to generate neurons for all cortical layers (Fig. 3B) [85, 86]. These results provide compelling evidence that environmental cues at different developmental stages are sufficient to program and reprogram the fate of either early or late neural progenitors, despite the fact that late IPCs are committed progenitors. Moreover, ablation of early-born deep-layer neurons prolongs the early neurogenic process and delays the switch to producing upper-layer neurons [87], suggesting that extrinsic signals arising from progeny cells control the temporal progression of progenitor cells. Previous studies have identified Wnt7, Ntf3, and TGFβ as feedback signals from nascent neurons to flip the temporal switch for neural progenitors and drive the early-to-late neurogenic transition [88–91]. Beyond these feedback signals, activation of Wnt signaling has also been reported to reprogram late NPCs into early multipotent progenitor cells, whereas signaling through Shh drives a subpopulation of neocortical NPCs to switch their fate from producing projection neurons to Gsx2^+^ IPCs that are committed to generate GABAergic neurons for the olfactory bulb (Fig. 3C) [92]. Thus, temporal restriction of neural progenitor competence may be regulated by age-dependent extrinsic cues in autocrine, paracrine, and endocrine manners.

Intrinsic temporal factors and extrinsic environmental cues may cooperate with each other to determine the temporal patterning of neural progenitors during brain development. Despite a lack of evidence, reciprocal interactions between intrinsic states and extrinsic cues can be easily envisaged to regulate transcriptional priming and epigenetic modification that ultimately lead to the generation of different neuronal subtypes in an invariant temporal order.

## Postmitotic Regulation of Neuronal Diversity

Following the postmitotic division of neural progenitors, the unspecialized nascent neurons are subjected to microenvironmental factors, wiring factors, and neural activity, as well as the well-established subtype-specific or state-specific TFs, and these contribute to further fate diversification. Here we provide a comprehensive introduction to these hypothetical mechanisms, which may raise many open questions yet to be answered.

**Fig. 4:**
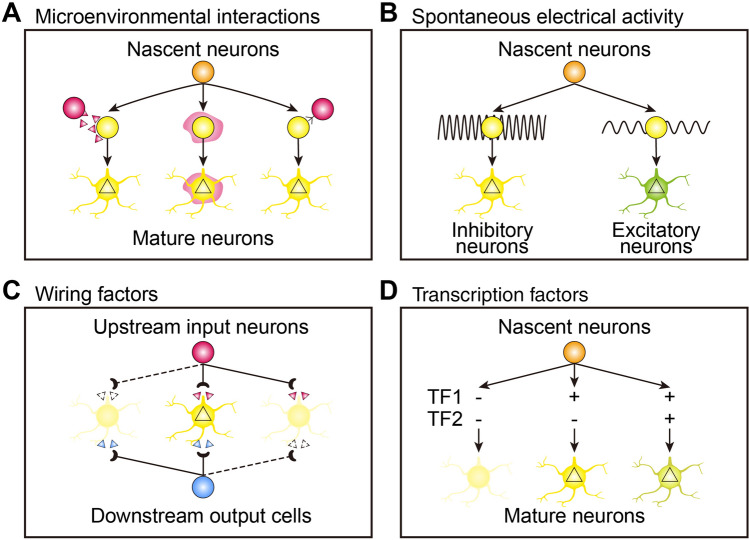
Primary regulatory mechanisms of neuronal diversification at the postmitotic level. **A** Microenvironmental factors, such as soluble factors, extracellular matrix, and other cells, reshape the identity of migrating nascent neurons. **B** Spontaneous electrical activity of different magnitudes determines the fate specification of unspecialized neurons. **C** The integration of nascent neurons into neural circuit sculpts their fate during maturation. **D** The stochastic expression of transcription factors at the postmitotic stage may diversify the fate of unspecialized neurons.

First, the interaction of migrating nascent neurons with microenvironmental factors *en route*, such as soluble factors, extracellular matrix, or other cells, may confer differential neuronal identities and increase neuronal diversity. Given the long-distance migration route, the cortical interneuron has been regarded as an excellent model in which to investigate whether the fate of unspecialized interneurons is shaped by different microenvironmental factors during their migration journey and after reaching their destination (Fig. 4A). Although the disordered lamination of excitatory projection neurons or lack of subcortical projection neurons in the cerebral cortex has been found to alter the positional identity of PV and SST neurons, their molecular identities seem not to be reshaped, as confirmed by recent studies showing the early fate determination of these interneurons [6, 7, 29, 30]. Notably, it has been suggested that specific neuronal subtypes, including CGE-derived interneurons (e.g., Vip and Id2 subsets) and many hypothalamic neuropeptidergic neurons, do not reach maturity at the transcriptomic level until postnatal stages, despite their entry into a postmitotic state during the early embryonic stage. These findings suggest the potential involvement of microenvironmental factors in shaping and diversifying the fate of neuronal precursors at the postmitotic level. However, the fate plasticity of unspecialized nascent neurons in different microenvironments and the critical extracellular factors remodeling neuronal fate remain to be explored by transplantation experiments, transdifferentiation assays, and high-throughput proteomic analysis.

Second, spontaneous electrical activity in the developing nervous system has been shown to endow nascent neurons with specific neurotransmitter identities before synaptic connection [93–95]. It is well characterized that spontaneous Ca^2+^ spike activity drives the neurotransmitter specification of unspecialized neurons in the developing *Xenopus* spinal cord, where neurons with a high frequency of Ca^2+^ spikes are specified into the GABAergic identity, while those with a low spike frequency adopt the glutamatergic and cholinergic phenotypes (Fig. 4B) [94]. Mechanistically, Ca^2+^ activity triggers a cell-autonomous signaling cascade by phosphorylating cJun and thereby represses the transcription of neurotransmitter selector gene Tlx3, which confers GABAergic rather than glutamatergic identity on unspecialized spinal neurons [93]. However, little is known about whether spontaneous neural activity switches neurotransmitter identity and confers fate plasticity to nascent neurons in the mammalian nervous system. It is notable that lowering neuronal excitability by Kir2.1-meidated hyperpolarization has been shown to shift the positional identity of two subsets of CGE-derived interneurons [96], which may open the possibility that spontaneous electrical activity regulates the region-specific differentiation of cortical interneurons.

Third, the unspecialized neurons reaching their permanent residence need to establish wiring with input neurons and output cells, which may induce neuronal fate specification and diversification. The anterograde signals from upstream input neurons and the retrograde cues from target regions are collectively referred to as wiring factors (Fig. 4C). In the developing somatosensory cortex, projection neurons in layer 4 receive mutually exclusive thalamocortical inputs from two thalamic nuclei conveying different sensory inputs and determine their fate postnatally [97, 98]. Interestingly, it has recently been found that distinct thalamocortical inputs specify the molecular and functional identity of layer 4 neurons by exerting sensory modality-specific controls, implicating a role of anterograde signals from input neurons in diversifying neuronal identities [8]. In parallel, extrinsic cues from target organs, such as skin and muscle, have also been suggested to retrogradely act on the axons of unspecialized sensory neurons and establish their identity during the development of the peripheral nervous system [9, 99]. As a well-characterized example, proprioceptive sensory neurons targeting muscles distributed along the D–V and proximodistal axes frequently acquire muscle-specific identity with discriminative marker genes. Genetic manipulation of the D–V feature of targeting muscles elicits a significant change in the gene expression profile of proprioceptive neurons, demonstrating an instructive role of retrograde signals from targeting cells in patterning neuronal subtype identity.

These fascinating mechanisms often reshape the features of postmitotic nascent neurons in different contexts and at different times, and thereby diversify neuronal identity. As neuronal subtype-specific TFs are frequently expressed at the postmitotic stage, the microenvironmental cues, neural activity, and wiring factors might determine the terminal status of unspecialized neurons by inducing the transcriptional activation of key TFs, which establish and maintain the identity of neurons (Fig. 4D). Nevertheless, the delicate molecular logic dictating these processes is largely unknown.

## Disruption of Lineage Specification Causes Brain Disorders

Genetic mutations and environmental insults may disrupt lineage development and impair neuronal diversification, which have been shown to predispose human beings to neurodevelopmental, neuropsychiatric, and neurodegenerative diseases [100–102]. Although previous studies have provided a wealth of knowledge on the molecular mechanisms of neural development, the genetic control of lineage specification in the developing brain remains underappreciated. In the cerebral cortex, Fezf2 is expressed in early cortical progenitors and deep-layer subcortical projection neurons [103], implying that Fezf2 mediates lineage fate determination. Genetic disruption of *Fezf2* indeed causes a complete loss of subcortical projection neurons, which instead adopt the fate of callosal projection neurons [31, 104]. Importantly, human genetic studies have also identified *Fezf2* as an autism candidate gene [105, 106], suggesting the involvement of the Fezf2-dependent cellular lineage in neurodevelopmental disorders. In the hypothalamus, Sim1 is strongly expressed in the anterior zone during early development and persists into mature neurons in the paraventricular nucleus [107]. Interestingly, Sim1 haploinsufficiency disrupts lineage specification by compromising the generation of a subset of neurons (e.g., oxytocin+ and vasopressin+ cells) and augmenting another subset of neurons, which may thereby lead to hyperphagic obesity [108, 109]. Patients carrying loss-of-function mutations in *SIM1* have also been reported to display obesity and a Prader-Willi syndrome-like phenotype [110]. Furthermore, environmental insults such as maternal inflammation, stress, malnutrition, smoking, alcohol drinking, and drug abuse also have a profound impact on fetal brain development. Although most maternal insults disturb cortical development without lineage bias, it has recently been demonstrated that early maternal inflammation specifically compromises the MGE-derived neuronal lineage, leading to a decline in the production of PV and SST interneurons [111]. Given the compelling evidence showing a deficiency in GABAergic interneurons in patients with mental illness, the selective vulnerability of the MGE-derived cell lineage to maternal insults may suffice to underlie the cognitive defects in affected offspring [101]. Taken together, these studies suggest that disrupted lineage specification caused by either genetic or environmental factors contributes to various neural diseases.

## Conclusion and Outlook

After decades of research, the current conceptual framework does not support a unifying strategy to generate a broad range of neuronal subtypes across different regions in the vertebrate nervous system. Instead, neural progenitors with different spatiotemporal codes deploy at least three general strategies to produce diverse neuronal subtypes, which may further diversify upon exposure to a different environment. Although different regions in the vertebrate nervous system adopt distinct strategies to produce neuronal diversity, the spatial and temporal patterning of neural progenitors seems to serve as an overarching principle that orchestrates lineage progression and neuronal diversification. Nevertheless, our understanding of how the spatial gradients of morphogens and the temporal progression of lineages are converted into such an extremely complex diversity of neural progenitors and neurons remains rudimentary. The nature of primary regulatory mechanisms that govern the self-enforcement of neural cell identity, the quantum effects of stochastic transcriptional noise, and temporal shifts of progenitor cell competence are also not clear. Among vertebrates, the evolution of the nervous system has been shown to drive the emergence of new progenitor types to increase neural complexity, but novel types of neural progenitor, such as outer radial glial cells, largely contribute to increased neuronal numbers but not neuronal diversity. Thus, it is fundamentally important to understand whether and how human-specific genes and genetic variants regulate the production of neuronal diversity across different brain regions.
